# A phenotypically supervised single-cell analysis protocol to study within-cell-type heterogeneity of cultured mammalian cells

**DOI:** 10.1016/j.xpro.2021.100561

**Published:** 2021-05-25

**Authors:** Kevin Chen, Kivilcim Ozturk, Ted Liefeld, Michael Reich, Jill P. Mesirov, Hannah Carter, Stephanie I. Fraley

**Affiliations:** 1 Department of Bioengineering, University of California, San Diego, La Jolla, CA 92093, USA; 2 Department of Medicine, Division of Medical Genetics, University of California San Diego, La Jolla, CA 92093, USA; 3 Bioinformatics and Systems Biology Program, University of California San Diego, La Jolla, CA 92093, USA; 4 Moores Cancer Center, University of California, San Diego, La Jolla, CA 92093, USA

**Keywords:** Bioinformatics, Cell culture, Cell isolation, Single Cell, Flow Cytometry/Mass Cytometry, Cell-based Assays, RNA-seq, High-Throughput Screening, Microscopy, Molecular Biology

## Abstract

Here, we describe a protocol combining functional metrics with genomic data to elucidate drivers of within-cell-type heterogeneity via the phenotype-to-genotype link. This technique involves using fluorescence tagging to label and isolate cells grown in 3D culture, enabling high-throughput enrichment of phenotypically defined cell subpopulations by fluorescence-activated cell sorting. We then perform a validated phenotypically supervised single-cell analysis pipeline to reveal unique functional cell states, including genes and pathways that contribute to cellular heterogeneity and were undetectable by unsupervised analysis.

For complete details on the use and execution of this protocol, please refer to Chen et al. (2020).

## Highlights

• Sorting of cells by the phenotype from 3D culture is achieved through photoconversion
• The photolabeling technique is adaptable to other systems, cells, and phenotypes
• Phenotypically supervised analysis reveals novel insights into cellular heterogeneity
• A GenePattern notebook facilitates phenotypically supervised scRNAseq analysis

## Graphical abstract

**Figure:**
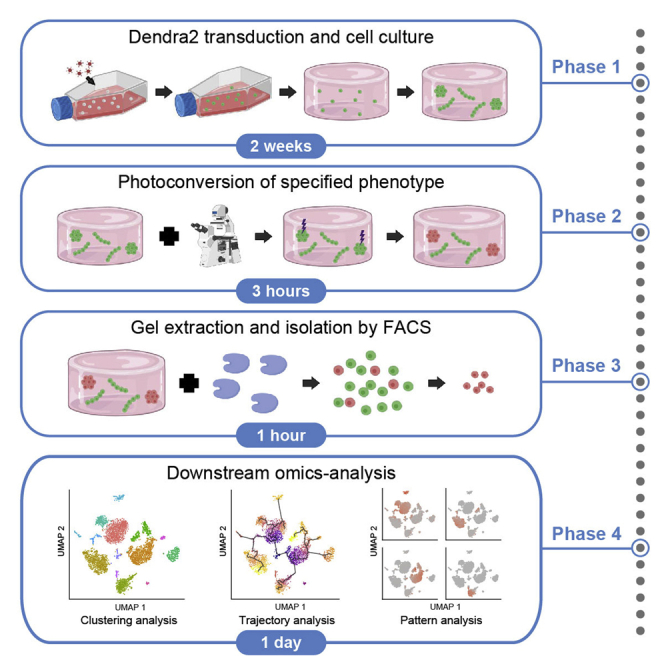

## Before you begin

### Introduction

Single cell transcriptomic analysis has deepened and advanced our understanding of developmental and disease biology in large part by revealing new cell types and new transcriptional states within cell types (Plasschaert et al., 2018; Suo et al., 2018; Jang et al., 2018). Yet, cellular states inferred from single cell RNA sequencing (scRNAseq) will not necessarily coincide with cellular behaviors, since these behaviors also depend heavily on environmental context (Chen et al., 2020). Since sequencing requires sample destruction and prohibits subsequent functional characterization of the cell states identified, it is not possible to subsequently verify whether inferred cell states accurately represent functional cell states.

On the other hand, cell biologists have observed and characterized a vast array of functional cell states within cell types, but the mechanisms underlying this heterogeneity often remain elusive. To bridge this gap between transcriptome-defined cell state and cell function, recent advances have integrated transcriptome measurements with cell electrophysiology (Cadwell et al., 2016), lineage tracing (Kester and van Oudenaarden, 2018), spatial information (Lein et al., 2017), multiple omics (Chappell et al., 2018), and genotype (Dixit et al., 2016; Jaitin et al., 2016). Others have focused on the challenge of isolating phenotypically distinct subpopulations for comparison using physical (Beri et al., 2020) or image-guided techniques (Konen et al., 2017). Still, the separation of cells by phenotype is non-trivial and is further complicated by the use of 3D culture models, which better recapitulate native physiology. Extraction of cells embedded in 3D culture presents challenges associated with low yields that limit the statistical power of comparisons and speed of processing that hinders the ability to capture transient cell states of interest in highly plastic cells. These experimental challenges translate into hurdles for computational analyses of within-cell-type heterogeneity, causing them to be under-developed or not well validated.

### Development of the protocol

To advance phenotype-based cell separation towards enabling statistically powered analyses, we have developed a protocol that enables (1) higher throughput fluorescent tagging of cells within 3D culture, (2) rapid release of cells from 3D culture and subpopulation enrichment using fluorescence-activated cell sorting (FACS) prior to scRNAseq, and (3) a validated analysis pipeline that distinguishes inferred cell states from phenotypically-supervised functional cell states. This method relies on the fluorescent tagging of the subpopulation of interest through photoconversion. Dendra2 was chosen as the photoconvertible protein, which can be transduced into any cell line, rendering them green-fluorescent. Upon exposure to 405 nm light, Dendra2 becomes red-fluorescent. Dendra2 was originally used to track intracellular protein movement, but we found that it could also be used as a general fluorescent tag to mark cells within 3D culture.

Using this protocol, we isolated subpopulations of cells that exhibit invasive versus non-invasive modes of collective migration. Subjecting these populations to transcriptomic sequencing followed by analysis of active gene expression programs revealed genes and pathways unique to each mode that were not detected by unsupervised analysis. Previously, we validated several of the differentially expressed genes with immunostaining and functional perturbation (Chen et al., 2020). This approach of using an agnostic fluorescent tag to perform phenogenomic sequencing is a powerful way to link functional output to transcriptomic data without the need for prior knowledge of biomarkers that define heterogeneous profiles.

The protocol presented here can be used for high-throughput separation of phenotypically heterogeneous cells in 3D culture based on any visual indicator, coupled with custom computational analysis to identify indicator-relevant genes and pathways. We describe a photoconversion-based platform that enables fluorescent tagging of visual phenotypes based on morphological characteristics. We include a streamlined 3D culture method, matrix digestion and FACS protocol, and an expanded data analysis procedure post scRNAseq composed of both supervised and unsupervised clustering, gene expression pattern detection and pseudotime trajectory analysis. The protocol thus provides an end-to-end strategy to characterize functional heterogeneity associated with visual cues.

We also provide a user-friendly notebook interface to automate the computational analyses, making them easy to apply to any scRNAseq data. Implementing multi-step bioinformatic pipelines for data processing and analysis can represent a barrier for reproducing analyses or applying them to new data sets. To address this, our computational workflows are encoded in GenePattern notebooks that provide well-documented and easily modifiable code. Furthermore, these notebooks can be run locally or online via the GenePattern server and are self-contained such that users do not need to install dependencies or third party software. New users can ensure understanding and reproducibility by running our dataset on the GenePattern notebooks as a test case.

### Comparison with other methods

Current methods developed for phenogenomic sequencing often require more stringent and less flexible methods of isolating the phenotype of interest (Cadwell et al., 2016; Jaitin et al., 2016; Dixit et al., 2016), and are limited to situations where established biomarkers already define the subpopulation to be isolated. Other agnostic labeling methods using confocal microscopy provide more flexibility but are often limited in throughput and scale (Konen et al., 2017). While lower yields are addressed by growth based amplification post-collection, this process may remove important regulatory signals originating from other cells or the extracellular matrix, and those that reset with cell divisions, hampering the ability to fully identify the cell states underlying the observed heterogeneity. The method we present here enables the tagging of thousands of cells with each run, providing roughly 10× more throughput and yield. Importantly, this provides enough sample material to negate the need for post-collection amplification for downstream studies.

Multiple bioinformatic tools have been developed for the processing of scRNAseq data into gene expression estimates and subsequent analysis to study within sample variation and infer distinct cellular states. Rather than develop a new pipeline, our protocol leverages existing analyses to extract additional information from scRNAseq by taking advantage of our phenotypic metadata. Current bioinformatic tools apply analyses of cell cycle, pseudotime trajectory (Cao et al., 2019; Trapnell et al., 2014; Kowalczyk et al., 2015), and gene pattern expression analysis (Fertig et al., 2010) agnostically to the transcriptomes of heterogeneous populations of cells. However, with our method, cells are sorted according to phenotype before scRNAseq, adding a new layer of data that is then used to supervise downstream bioinformatic analyses and inform interpretations. The addition of phenotype labels enables the refinement of results from computational analysis and generates new knowledge that cannot be gained by computational analysis alone. The scRNAseq analysis on the basis of phenotype labels results in a more functionally relevant gene set that discriminates between different phenotypes and accurately identifies key processes, as verified by functional studies. Compared to unsupervised analysis, the use of phenotypic metadata detected a more selective gene set (from 528 to 178), and importantly, detected 70 unique differentially expressed genes (DEGs) that were not detected by unsupervised clustering. Here, we also show that supplementing cell cycle, pseudotime, and gene expression pattern analysis with phenotypic metadata enables new cell states and state transition trajectories to be identified. Our protocol illustrates the power of a supervised, functionally informed computational analysis and enables a more direct approach to investigating the mechanisms underlying cellular heterogeneity.

### Cloning of Dendra2 into lentiviral vector

**Timing: 1 week**

1. Amplify the Dendra2-Lifeact vector using PCR with the custom primers. Thermocycling conditions are listed in Table 1. The recipe for the PCR mixture is provided in Table 2. Phusion and the 5× HF buffer, and dNTPs are all provided in the Phusion High-Fidelity PCR Kit. (https://www.thermofisher.com/document-connect/document-connect.html?url=https%3A%2F%2Fassets.thermofisher.com%2FTFS-Assets%2FLSG%2Fmanuals%2FMAN0013363_Phusion_HiFi_PCR_Kit_UG.pdf&title=VXNlciBHdWlkZTogUGh1c2lvbiBIaWdoLUZpZGVsaXR5IFBDUiBLaXQ=)

**Table 1:** Thermocycling conditions

Steps	Temperature	Time	Cycles
Initial Denaturation	98°C	30 s	1 cycle
Denaturation	98°C	10 s	30 cycles
Annealing	68°C	20 s	
Extension	72°C	20 s	
Final Extension	72°C	5 min	1 cycle

**Table 2:** PCR recipe

Component	Final concentration
5× HF buffer	1×
dNTPs	0.2 mM
Forward Primer	0.2 μM
Reverse Primer	0.2 μM
Phusion	0.02 U/μL
Dendra2 vector	0.2 μg/μL
PCR Water	Fill for remaining volume
2. Digest the pSin plasmid using EcoRI and SpeI.
3. Run the digested plasmid, along with an undigested control, on a 1.5 wt% agarose gel.
4. Check for successful digestion, and gel purify the digested backbone using QIAQuick gel extraction kit. (https://www.qiagen.com/us/resources/resourcedetail?id=95f10677-aa29-453d-a222-0e19f01ebe17&lang=en)
5. Clone the Dendra2-Lifeact fragment into the pSin backbone using T4 Ligase at an insert:vector ratio of 3:1.
6. Transform the plasmid into competent DH5a for amplification.
7. Harvest the lenti-Dendra2 vector using the Promega miniprep kit. (https://www.promega.com/products/nucleic-acid-extraction/plasmid-purification/wizard-plus-sv-minipreps-dna-purification-systems/?catNum=A1330#protocols)
8. Verify the sequence through sequencing with the custom primers.

### Production of lentiviruses

**Timing: 3-4 days**

9. Culture HEK293T cells in a 6-well plate until 70%–80% confluency.
10. Transfect the cells 16–24 h after plating with the lenti-Dendra2 plasmid along with lentiviral packaging (psPAX2) and envelope (pMD2.G) vectors using the Lipofectamine 3000 kit. (https://www.thermofisher.com/document-connect/document-connect.html?url=https%3A%2F%2Fassets.thermofisher.com%2FTFS-Assets%2FLSG%2Fmanuals%2Flipofectamine3000_protocol.pdf&title=TGlwb2ZlY3RhbWluZSAzMDAwIFJlYWdlbnQgUHJvdG9jb2wgKEVuZ2xpc2gp) For the DNA component, add approximately 1 μg of each of the 3 plasmids, per well. Add the transfection mix, which contains the plasmids, Lipofectamine, and P3000 drop-wise to the cells cultured in complete medium I and gently swirl the plate to mix.
11. Twenty-four hours after transfection, replace the medium with fresh medium. Check the cells, using a fluorescence microscope to determine transfection efficiency.
12. Harvest the virus-containing medium on the 3rd day after transfection. Check the cells again, using a fluorescence microscope to determine virus production efficiency. Filter the medium using the 0.45 μm filter to remove cell debris. Collect the virus in 1.5 mL microcentrifuge tubes and store at -80°C for long term storage or use immediately for transduction.

## Key resources table

**Table:** 

REAGENT or RESOURCE	SOURCE	IDENTIFIER
**Bacterial and virus strains**		

DH5α competent cells	Thermo Fisher	18258012

**Chemicals, peptides, and recombinant proteins**		

Gentamicin	Life Technologies	15750060
Rat tail collagen I	Fisher Scientific	CB354249
Sodium hydroxide	Fisher Scientific	S318-500
(4-(2-Hydroxyethyl)-1-piperazineethanesulfonic acid) HEPES, Free Acid	Millipore Sigma	5310-OP
Sodium bicarbonate	MP Biomedicals	02119484783
Polyethylene glycol	Sigma-Aldrich	P5413-500G
Kanamycin sulfate	Fisher Scientific	11-845-024
Ampicillin	Fisher Scientific	BP1760-5
ECORI	NEB	R0101S
SPEI	NEB	R0133S
T4 ligase	NEB	M0202S
Collagenase	Sigma-Aldrich	C0130
Bovine serum albumin (BSA)	Fisher Scientific	BP671-10
EDTA (500 mM)	BioPioneer	MB1010
DMEM	Gibco	11995065
FBS	Corning	35-010-CV
PBS	Gibco	10010023

**Critical commercial assays**		

Chromium Chip B Single Cell Kit	10x Genomics	1000154
Chromium i7 Multiplex Kit	10x Genomics	120262
Chromium Single Cell 3’ Library & Gel Bead Kit v3	10x Genomics	1000092
Chromium Single Cell 3’ Library Construction Kit v3	10x Genomics	1000078
Lipofectamine 3000 Kit	Thermo Fisher	L3000008
Promega Miniprep Kit	Promega	A1330
QIAQuick Gel Extraction Kit	QIAGEN	28704
Phusion High-Fidelity PCR Kit	Thermo Fisher	F553S

**Deposited data**		

scRNAseq data	Gene Expression Omnibus	GSE158844

**Experimental models: cell lines**		

MDA-MB-231	ATCC	HTB-26
Lenti-X 293T	Takara Bio	632180

**Oligonucleotides**		

Forward primer (5’ TAAGCAACTAGTGGTTTAGTG AACCGTCAGA 3’)	IDT	N/A
Reverse primer (5’ GGTGCTTAGAATTCGTAAAAC CTCTACAAATGTGG 3’)	IDT	N/A

**Recombinant DNA**		

pSin-EF2-Nanog-Pur	Addgene	16578
Dendra-2-Lifeact-7	Addgene	54694
psPAX2	Addgene	12260
pMD2.G	Addgene	12259

**Software and algorithms**		

Seurat v3.1.1	Satija Lab	RRID: SCR_007322
Monocle3 v0.2.1	Trapnell Lab	RRID: SCR_018685
PANTHER v15.0	pantherdb.org	RRID: SCR_004869
CoGAPS v3.4.1	Bioconductor	RRID: SCR_001479
Cell Ranger v3.0.2	10x Genomics	RRID: SCR_017344
Nikon Elements AR v4.51.00	Nikon	RRID: SCR_014329
DESeq2 v1.24.0	Bioconductor	RRID: SCR_015687
GenePattern notebook server	http://notebook.genepattern.org	RRID: SCR_015699

**Other**		

0.45 μm Sterile filter	VWR	28137-938
6-Well plate	Corning	353046
48-Well plate	Corning	353078
BD Influx Sorter	BD Bioscienes	646500
Nikon TiE Inverted Microscope	Nikon	N/A
LU-N4	Nikon	N/A
Galvo Miniscanner	Nikon	N/A

## Materials and equipment

### Reagent setup

#### Complete medium I

For cell line expansion, prepare complete medium I by supplementing Dulbecco’s Modified Eagle Medium (DMEM) (450 mL) with Fetal Bovine Serum (FBS) (50 mL), and Gentamicin (500 μL). The details of the recipe can be found below. Sterile filter and store at 4°C until needed. Before use, warm up in a water bath (37°C). Complete medium I can be stored at 4°C for up to 6 months.

**Table:** Complete medium I recipe

Reagent	Final concentration (vol %)	Amount
DMEM	∼90% (v/v)	450 mL
FBS	∼10% (v/v)	50 mL
Gentamicin	10 μg/mL	500 μL

#### Fluorescence-activated cell sorting (FACS) buffer

Mix 0.2 g BSA, 20 μL of Ethylenediaminetetraacetic acid (EDTA), and 50 mL of PBS. The details of the recipe can be found below. Sterile filter and store at 4°C until needed. FACS buffer can be stored at 4°C for 4 months.

**Table:** FACS buffer recipe

Reagent	Final concentration	Amount
BSA	0.4%	0.2 g
EDTA (500 mM)	0.2 mM	20 μL
PBS	-	50 mL

#### Reconstitution buffer (RB)

Mix 110 mg NaHCO_3_, 240 mg HEPES free acid, and 5 mL nanopure water to make a stock solution. Sterile filter, aliquot, and store at −20°C until needed. RB can be stored at −20°C for 1 week.

**Table:** 

Reagent	Final concentration	Amount
NaHCO_3_	0.26 M	110 mg
HEPES free acid	0.2 M	240 mg
Nanopure water	-	5 mL

#### Polyethylene glycol (PEG)

Mix PEG with PBS to make a 100 mg/mL stock solution. Sterile filter, aliquot, and store at −20°C until needed. PEG can be stored at −20°C for 3 weeks.

#### NaOH

Mix NaOH with nanopure water to make a 1N stock solution. Sterile filter and store at −20°C until needed. NaOH can be stored at −20°C for 6 months.

#### Gel digestion buffer

Mix 10 mg of collagenase with 1 mL of PBS. Sterile filter and store at −20°C until needed. Gel digestion buffer can be stored at 4°C for 6 months.

### Equipment setup

#### Photoconversion

Before you begin, make sure to calibrate the laser with your galvo scanner. You will need to determine the appropriate settings for some key simulation parameters, such as dwell time and laser power. Optimize these parameters to obtain high post-photoconversion fluorescence but minimize exposure to avoid phototoxicity. Observe your cells post-photoconversion to confirm their behavior is similar to non-photoconverted counterparts. As a general guideline, photoconverted cells should display 3× higher red fluorescence intensity compared to the background noise when observed using microscopy for successful isolation using FACS. In our setup, each collective cell structure was exposed to approximately 1 mJ of light.

#### FACS

Ensure that the 405 nm laser line on the machine can be turned off, as this can result in photoconversion during flow sorting. Ensure that the equipment contains the appropriate lasers and filters to capture both the native and photoconverted fluorescent states to ensure enrichment of the desired population. A machine that can support chilled sorting is preferred to maintain viability of the cells.

## Step-by-step method details

The protocol below describes the specific steps for interrogating heterogeneous collective migration phenotypes of MDA-MB-231 cells in a 3D collagen matrix. However, this protocol can be used with other cell lines and other culture platforms and is easily adaptable to other biological contexts that can be visually defined.

**Figure 1:**
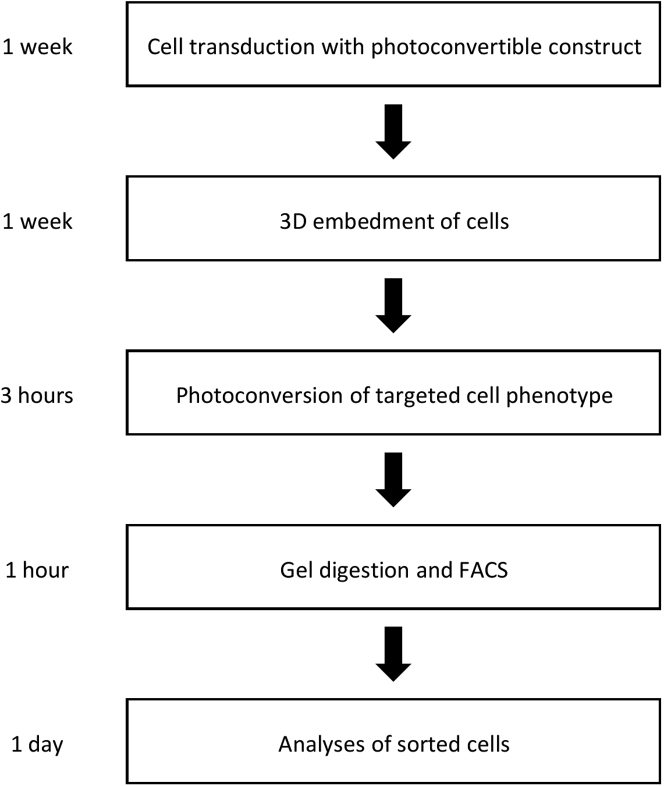
Protocol flowchart The major components of the protocol are: transduction of cells with a photoconvertible construct, 3D culture of cells, photoconversion, gel digestion and FACS, and downstream analyses. Time needed to complete these steps is shown on the left.

The experimental workflow and timing of the procedures is shown in Figure 1, which depicts the different stages of the Procedure. In broad terms, the Procedure consists of five main sections, which are detailed below.

***Note:*** It is important to verify that the biological heterogeneity being studied is not altered through expression of the photoconvertible protein or the photoconversion process. Cells expressing the photoconvertible protein should be compared to wild-type cells to ensure the same modes of heterogeneity exist and at similar frequencies. In addition, to confirm that the photoconversion process does not significantly alter gene expression, a non-labeled population can be compared to the sorted populations through gene expression assays to demonstrate minimal difference. In particular, genes associated with responses to light should not be significantly differentially expressed between labeled and unlabeled populations.

***Note:*** Proper controls must also be used to ensure collection of purified photoconverted populations. A non-photoconverted sample exhibiting equivalent biological heterogeneity should be used to gate the baseline fluorescence of the photoconverted channel. To ensure purity of the sample, a strict gate should be applied where all cells collected express higher fluorescence in the photoconverted channel compared to the negative control. Users can adjust the strictness of this gate to their application based on the demands for the purity of their enriched population.

### Viral transduction of Dendra2 into MDA-MB-231

**Timing: 4-5 days**

**CRITICAL: Choice of cell line**- Successful transduction of a photoconvertible protein into the experimental cell line is a foundation of this technique. Thus, selection of a cell line amenable to transduction is critical. While we present a method of lentiviral transduction to induce expression of Dendra2 in our protocol, other methods of induced gene expression and other photoconvertible proteins may be used as well.

1. Culture MDA-MB-231 cells in a 6-well plate until 70%–80% confluency. We recommend working with cells below 20 passages.
2. Aspirate the media, wash the cells once with PBS, and replace with new growth media.
3. Add 75 μL of collected lenti-Dendra2 dropwise to the well. Gently swirl to mix.
4. Monitor transduction efficiency through fluorescent microscopy. It may take 2-3 days before cells start to fluoresce.

See troubleshooting problem 1

5. Passage cells into larger flasks to prepare for purification through FACS. Using a wild-type control, gate for the cells expressing above background levels of green fluorescence. You may choose to collect only the cells that have the highest fluorescence for ease of identification for downstream experiments.

### 3D culture of MDA-MB-231 Dendra2 (MDA-Dendra)

**Timing: 1 week**

**Culturing platform:** This protocol separates cells undergoing distinct collective migration behaviors within 3D culture, specifically in 3D type I collagen (COLI). For the end user, the biophysical properties of the environment, including choice of material, should be adapted according to the type of heterogeneity being studied. Tissue specific studies, for instance, may need to be matched with particular extracellular matrix proteins at specific densities and stiffnesses to ensure biological relevance. Separation of the photoconverted cell population in our system requires the use of collagenase and trypsin, which may destroy surface markers. If surface proteins need to be preserved for downstream applications, enzymes with less disruptive mechanisms can be used. An alternative approach would be the use of culture systems that enable non-enzymatic cell retrieval strategies, such as Matrigel or engineered synthetic hydrogels.

6. Thaw out RB, NaOH on ice.
7. Place a 48-well plate in the incubator to preheat to 37°C .
8. Passage MDA-Dendra and count the cells using a hemocytometer. Keep cells on ice.
9. Calculate the amount of reagents required to make the 3D collagen hydrogel. Table 3 displays sample calculations for making a 2.5 mg/mL collagen + 10 mg/mL PEG hydrogel, with 50,000 cells/mL embedded. Adjust calculations as necessary.
  **CRITICAL:** The following steps must be performed quickly and carefully. Carefully mix the solutions at every step and do not introduce bubbles into the solution. If bubbles form, start over as the architecture of the hydrogel will not be homogenous. Steps must be performed quickly, and reagents kept as cold as possible to prevent polymerization before the gel solution is incubated at 37°C .

**Table 3:** Collagen gel synthesis calculations

Stocks	Desired concentration or volume	Final volume
Collagen: 9.00 mg/mL	2.5 mg/mL	2.5∗0.25/9.00 = 69.4 μL
PEG: 100 mg/mL	10 mg/mL	10∗0.25/100 = 25 μL
NaOH	6.25% ∗ volume Collagen	0.0625∗69.4 = 4.34 μL
RB	Remaining Volume / 2	(250-69.4-25-4.34)/2 = 75.6 μL
Cells (200,000 cells/mL)	50,000 cells/mL	50∗0.25/150 = 62.5 μL
Media	Top off until gel volume is reached	250-69.4-25-4.34-75.6-62.5 = 13.2 μL

Total desired volume of gel: 200 μL. Make 250 μL to account for reagent loss during pipetting.

**Figure 2:**
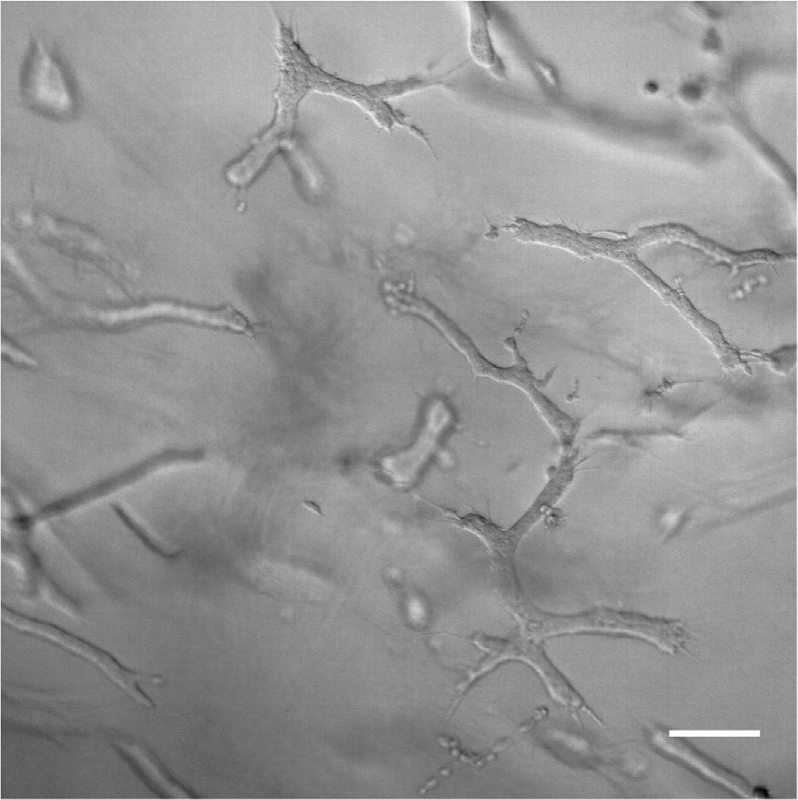
MDA-MB-231 after 1 week of culture in 3D collagen Representative bright field image (15×) of MDA-MB-231 cells grown in 3D collagen for 7 days to illustrate appropriate cellular density and morphologies. Scale bar, 100 μm.

10. Place the collagen, media, and an empty 1.5 mL microcentrifuge tube on ice.
11. Add the reagents in the empty microcentrifuge tube in the following order: cells, media, PEG, RB, Collagen, NaOH. In between each addition, pipette mix the components at least 10×.
12. Immediately pipet the gel solution into the preheated well plate and incubate at 37°C for 30 min.
13. After 30 min, perform 3 washes of 1× PBS for 5 min each. The amount of PBS to pipette on top of the gel is the same as the gel volume.
14. After the last wash, aspirate the PBS and add growth media on top of the gel. The amount of growth media to pipette on top of the gel is the same as the gel volume.
15. Incubate the 3D culture for a week, changing media every 2-3 days to keep the cells viable (Figure 2).

### Tagging of collective cell phenotypes by photoconversion

**Timing: 2**–**3 h**

The choice of imaging strategy is not limited to the specific microscopes and settings presented in this protocol. However, the imaging setup presented is specifically designed to achieve high throughput photoconversion, and as such consists of a widefield microscope. The imaging system must be equipped with a laser line of wavelength suitable for photoconversion, a galvanometric scanner, and climate control (temperature, humidity, and CO_2_). The choice of lenses should be optimized to focus the laser light as much as possible to minimize off-target photoconversion, while also taking into consideration the physical size of the attributes used to determine the modes of heterogeneity being studied and maximizing throughput (subpopulation photoconversion).

**Figure 3:**
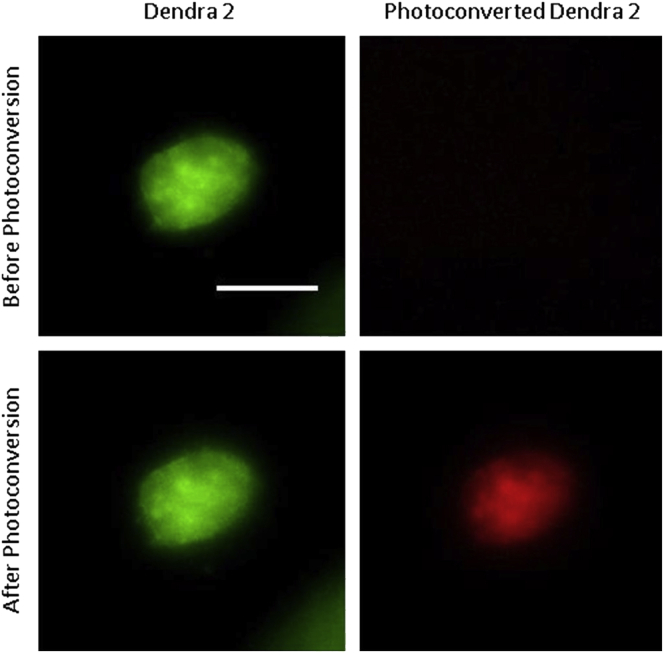
MDA-MB-231 before and after photoconversion Representative fluorescent images of MDA-MB-231 cells grown in 3D collagen to illustrate appropriate fluorescence levels to indicate successful photoconversion. After photoconversion, red fluorescence should be abundant in the photoconverted cells (bottom). Scale bar, 50 μm.

16. Transfer the collagen gel to a glass-bottom dish. Add enough media to keep the gel hydrated, but not so much that the gel will float in solution or move around in the dish.
17. Transfer the glass-bottom dish to a fluorescent microscope stage.
18. In the Bruker miniscanner panel, set the dwell time to 300 us and the 405 nm laser power to 25%.
19. Calibrate the galvanometric scanner.
20. Using a 20× lens, identify the cells you would like to photoconvert.
21. Verify that other cells are not within 10 μm in x-y and not within 200 μm in z.
22. Draw an ROI around the cells you would like to photoconvert. Right-click and select to use ROI as a Stimulation ROI.
23. In the Bruker miniscanner panel, click Stimulate.
24. Repeat steps 20–23 until all cells of interest within the hydrogel have been photoconverted (Figure 3).

See troubleshooting problem 2

### Gel extraction and FACS sorting of photoconverted cells

**Timing: 1-2 h**

Proper digestion and preparation of cells into a single cell suspension is crucial for the efficient collection of the fluorescently tagged population. We have minimized the processing time to preserve the transcriptional signature as much as possible. In our case, since we culture our cells in a COLI hydrogel, we chose to use collagenase as our matrix digestion enzyme. 3D cultures using other materials should use their respective appropriate depolymerization strategy and be optimized for a short processing time while minimizing adverse effects on the cells. Since we study collective phenotypes, we also had an incubation phase with trypsin followed by straining to further dissociate the cells. This may not be necessary in other cases where cells are less adherent to each other after gel digestion. We describe a standard buffer for FACS that was amenable to the survival of our cells. Cells that cannot survive in this basic flow sorting buffer may require other supplements or growth media while sorting.

The flow cytometer to be used must have the proper lasers and filters to detect the emission spectrum of the photoconvertible protein, in its native and photoconverted state. The equipment we use allows for stringent gating to ensure the collection of a phenotypically pure population. Users can adjust the gate depending on the stringency of their experiment. The flow sorter we use comes equipped with liquid chilling to help preserve our sample, although this may not be necessary in all cases.

25. Transfer the collagen gel to a 1.5 mL microcentrifuge tube.
26. Add 50 μL of 10 mg/mL collagenase to the gel.
27. Use a P1000 pipette tip to gently mash and mechanically disrupt the gel.
28. Incubate the gel in the water bath at 37°C for 5 min.
29. Mix the solution further with the P1000 pipet tip. The entire solution should be pipetted up and down within the pipette tip. Minimize the introduction of bubbles as much as possible.
30. Incubate the gel in the water bath at 37°C for 5 min.
31. Mix the solution with a P200 pipette tip. The entire solution should be pipetted up and down within the pipette tip. Minimize the introduction of bubbles as much as possible.
32. Incubate the gel in the water bath at 37°C for 5 min.
33. Centrifuge the solution at 400 × *g* for 4 min.
34. Discard the supernatant, add 50 μL of 0.25% trypsin, and resuspend the pellet.
35. Incubate in the water bath at 37°C for 5 min.
36. Mix the solution with a P200 pipette tip.
37. Incubate in the water bath at 37°C for 5 min.
38. Centrifuge the solution at 400 × *g* for 4 min.
39. Remove the supernatant and resuspend the pellet with an ice-cold FACS buffer.
40. Strain the cells prior to FACS.
41. Use forward and side scatter density plots to exclude debris.
42. Use un-photoconverted controls to set gates for red-fluorescence.
43. Collect cells expressing red-fluorescence higher than gate (Chen et al., 2020).

See troubleshooting problem 3

### Transcriptomic sequencing

**Timing: 2 days - 1 week**

We use the 10× 3’ v3 Gene Expression kit to prepare our samples for scRNAseq. However, users can choose to use other platforms depending on sample size and specific needs for sequencing coverage. Sequencing was performed on a Hiseq4000 to achieve a depth of at least 25,000 reads/cell. However, sequencing depth can be adjusted depending on the user’s needs and budget.

44. Prepare cell samples for scRNAseq using the 10× 3’ v3 Gene Expression kit.
45. Perform quality control on libraries using TapeStation.
46. Sequence the libraries using an Illumina Hiseq 4000.
47. Use the 10× genomics Cell Ranger pipeline to align reads to the appropriate reference genome. Here we use GRCh38.
48. Check the outputs to ensure that the sequencing depth was at least 20,000 reads per cell. Sequence the libraries more if needed.

### scRNAseq processing

**Timing: varies depending on computational resources and dataset sizes, ∼1-2 h on the GenePattern server**

We perform differential expression analysis using Seurat v3.1.1 using standard pre-processing protocols as listed on their documentation. Depending on the type of biological heterogeneity being studied, gene expression data may need to be further filtered or normalized.

49. Import dataset into Seurat (Stuart et al., 2019).

See troubleshooting problem 4

50. Filter the counts matrix to remove genes expressed in fewer than 3 cells. This parameter can be increased if desired.
51. Discard cells expressing too few or too many genes. Too few genes may indicate that the cell membrane has been breached and contents leaked. Too many may indicate cell doublets or other artifacts. These parameters must be adjusted to the specific dataset. Here we remove cells expressing fewer than 2000 genes or more than 6000 genes.
52. Discard cells that have over 20% of reads aligned to mitochondrial genes. This parameter can be decreased for more stringent filtering.
53. Normalize the counts matrix. We normalized to the default scale factor of 10,000.
54. Identify the top variable genes to be used for downstream analysis. Here we selected the default of 2000 genes.
55. Apply a linear transformation on the count matrix to scale and center expression of each gene.
56. Assign cell cycle scores to each cell based on its expression of G2/M and S phase markers and apply a linear model to regress out effects of cell cycle heterogeneity. The same approach could also be used for batch effect removal if simultaneously analyzing multiple datasets.
57. Perform linear dimensionality reduction (PCA) on the scaled data using the most variable genes.
58. Perform a graph-based clustering approach by first determining the nearest neighbors of each cell in the PCA space. Use the top principal components that explain the most variance in the dataset. We used the first 20 principal components.
59. Then cluster cells by applying a modularity optimization algorithm that iteratively groups cells together. A resolution parameter determines the number of clusters obtained. We specified a resolution of 0.05 to obtain 2 clusters based on our expectation that two phenotypes exist within our isogenic photolabeled cells. A higher resolution can be set up to achieve more clusters.
60. Use UMAP, a non-linear dimensionality reduction technique, to visualize the clusters where similar cells are placed together in low-dimensional space (Becht et al., 2018).
61. Apply Fisher’s Exact test to evaluate the enrichment or depletion of cell cycle phases in each cluster or phenotype. An odds ratio (OR)>1 indicates enrichment while an OR<1 indicates depletion.

### Differential expression analysis

**Timing: ∼1 h on the GenePattern server**

62. Identify positive and negative markers of each cluster or phenotype by applying the DESeq2 algorithm, which uses a negative binomial distribution (Love et al., 2014).
63. Perform multiple testing corrections using the Benjamini & Hochberg method to control the false discovery rate. Select differentially expressed genes for each cluster with adjusted p-value lower than 0.05.
64. Generate an expression heatmap to visualize the differential expression of these genes across clusters.

### Gene Ontology (GO) term over-representation analysis

**Timing: ∼30 min**

65. Determine GO terms that are over-represented in the positive markers for each cluster by inputting the list of genes to the PANTHER classification system (http://www.pantherdb.org) (Mi et al., 2019), and choosing Homo sapiens as reference organism and GO biological process complete annotation data set.
66. Choose Fisher’s Exact test as the statistical test and allow for multiple testing correction via the Benjamini & Hochberg method to control for False Discovery Rate (FDR). Only select significant terms with FDR<0.05.
67. Gene set statistics (p-values, gene set sizes and overlapping gene counts) can be exported from PANTHER and imported into Python to investigate processes of interest and generate visual summaries such as a bar plot where gene sets are ranked based on p-values.

### Pseudotime trajectory analysis

**Timing: ∼1 h on the GenePattern server**

68. Convert the processed Seurat object to a Monocle3 (Cao et al., 2019) object.
69. Learn the trajectory graph by fitting a principal graph within each partition.
70. Choose the roots of the trajectory representing the beginning of the biological process to order the cells according to their progress along the trajectory.
71. Plot the progress through pseudotime trajectory on UMAP plots colored by cell cycle phase, pseudotime, clusters, or expression of marker genes.

### Gene expression pattern analysis

**Timing: ∼3–4 h on the GenePattern server**

72. Extract the expression data for the top variable genes from the processed Seurat object as a matrix.
73. Apply Coordinated Gene Association in Pattern Sets (CoGAPS) (Fertig et al., 2010), a Nonnegative Matrix Factorization algorithm, on the matrix dataset with default parameters.

See troubleshooting problem 5

74. Extract the matrix of sample weights for each pattern learned by CoGAPS.
75. Visualize the patterns on UMAP plots using sample weights to evaluate associations with a particular cluster or phenotype.
76. Analyze the significance of the association using a Kruskal-Wallis rank-sum test.
77. Generate a sample weights by cells heatmap to visualize the patterns to further evaluate associations with a particular cluster or phenotype.

## Expected outcomes

As outlined in the protocol above, our approach comprises four major components: transduction of cells with Dendra 2, photoconverting the specified phenotypes of interest in a 3D culture platform, isolation of that phenotype by flow sorting, and downstream bioinformatic analysis. By implementing the procedures described in our protocol, one can readily use the approach of photoconversion to functionally isolate any phenotype that can be described by visual characteristics. While the end user will have to adapt analysis strategies to their specific contexts, we anticipate that the use of our downstream bioinformatic pipeline should enable the identification of potential biomarkers that regulate their system and inform further validation experiments.

**Figure 4:**
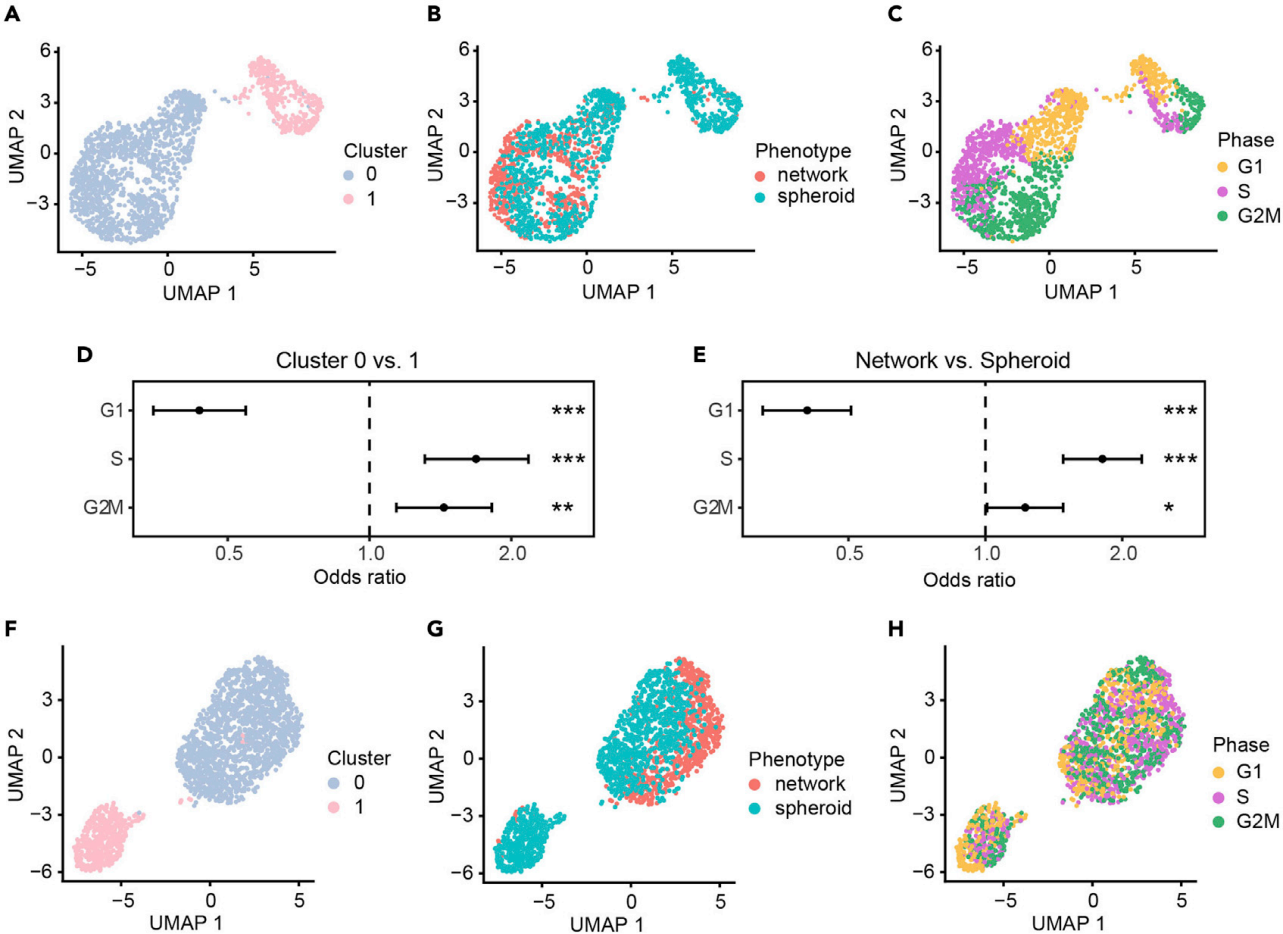
Comparative analysis of unsupervised clusters versus phenotypic labels (steps 49–61) (A–C) UMAP plots colored by (A) unsupervised clusters, (B) phenotypic labels, and (C) cell cycle phases. (D and E) Cell cycle phase distribution in (D) network vs. spheroid phenotypically labeled cells, or in (E) unsupervised clusters. Odds ratios (OR) and 95% confidence intervals of each cell cycle phase (G1, S and G2M) to be prevalent in (D) network vs. spheroid labeled cells or in (E) unsupervised clusters 0 vs. 1 using Fisher’s exact test are shown (∗p<0.05, ∗∗p<0.01, ∗∗∗p<0.0001). For (D) OR>1 means network cells are more prevalent in the indicated cell cycle phase compared to spheroid cells, and for (E) OR>1 means cells in cluster 0 are more prevalent in the indicated cell cycle phase compared to cells in cluster 1. (F–H) UMAP plots colored by (F) unsupervised clusters, (G) phenotypic labels, and (H) cell cycle phases, after regression of cell cycle phase effects.

Cells undergoing collective migration as invasive networks or spheroids were labeled, sorted, and subjected to scRNAseq analysis. After preprocessing, the first step of the analytic pipeline provides UMAP plots with cells annotated according to unsupervised clustering (Figure 4A), phenotypic category (Figure 4B) and cell cycle state inferred from canonical markers (Figure 4C). Because correlation between clusters may not be visually apparent, the analysis includes a statistical test for enrichment of cell cycle states. In this case, we found a statistically significant difference in cell cycle phase distribution between cells in cluster 0 vs. 1 (Chi-squared test p-value=3.43e-13), and between phenotypically labeled cells (Chi-squared test p-value=5.64e-17). Plotting the odds ratios associated with each cell cycle phase shows the relative enrichment of one cell cycle phase versus all others within a particular category of cells (ORs obtained by Fisher’s Exact test; Figures 4D and 4E). Cells of cluster 0 on average are significantly more prevalent in S (OR=1.68, p-value=2.57e-05) and G2M phases (OR=1.43, p-value=0.0017), and less prevalent in G1 phase (OR=0.43, p-value=2.08e-13) compared to cells in cluster 1 (Figure 4D). Network cells are significantly more enriched in S (OR=1.80, p-value=2.76e-09) and G2M phases (OR=1.22, p-value=0.038), but are depleted in G1 phase (OR=0.40, p-value=4.19e-17) compared to spheroid cells, suggesting that network cells are more proliferative compared to spheroid cells (Figure 4E).

In order to study gene regulation that is not associated with the proportion of cells in each group at different phases of the cell cycle, the pipeline next regresses out cell cycle effects. New UMAP visualizations are generated after this step. Here we note that the lack of correlation between the unsupervised clusters (Figure 4F) and the phenotypic labels (Figure 4G) remains, but there is now clearer separation between network and spheroid labeled cells that are clustered together in cluster 0. Cells in cluster 1 remain primarily composed of spheroid labeled cells (Figures 4F and 4G, Table 4). Cell cycle labels are now more evenly distributed, confirming removal of cell cycle effects through this procedure (Figure 4H).

**Table 4:** Distribution of supervised phenotypic labeled cells across unsupervised clusters

Unsupervised cluster label	Supervised phenotype label	Number of cells
0	Spheroid	848
1	Spheroid	416
0 & 1	Spheroid	1264
0	Network	693
1	Network	25
0 & 1	Network & Spheroid	1957

**Figure 5:**
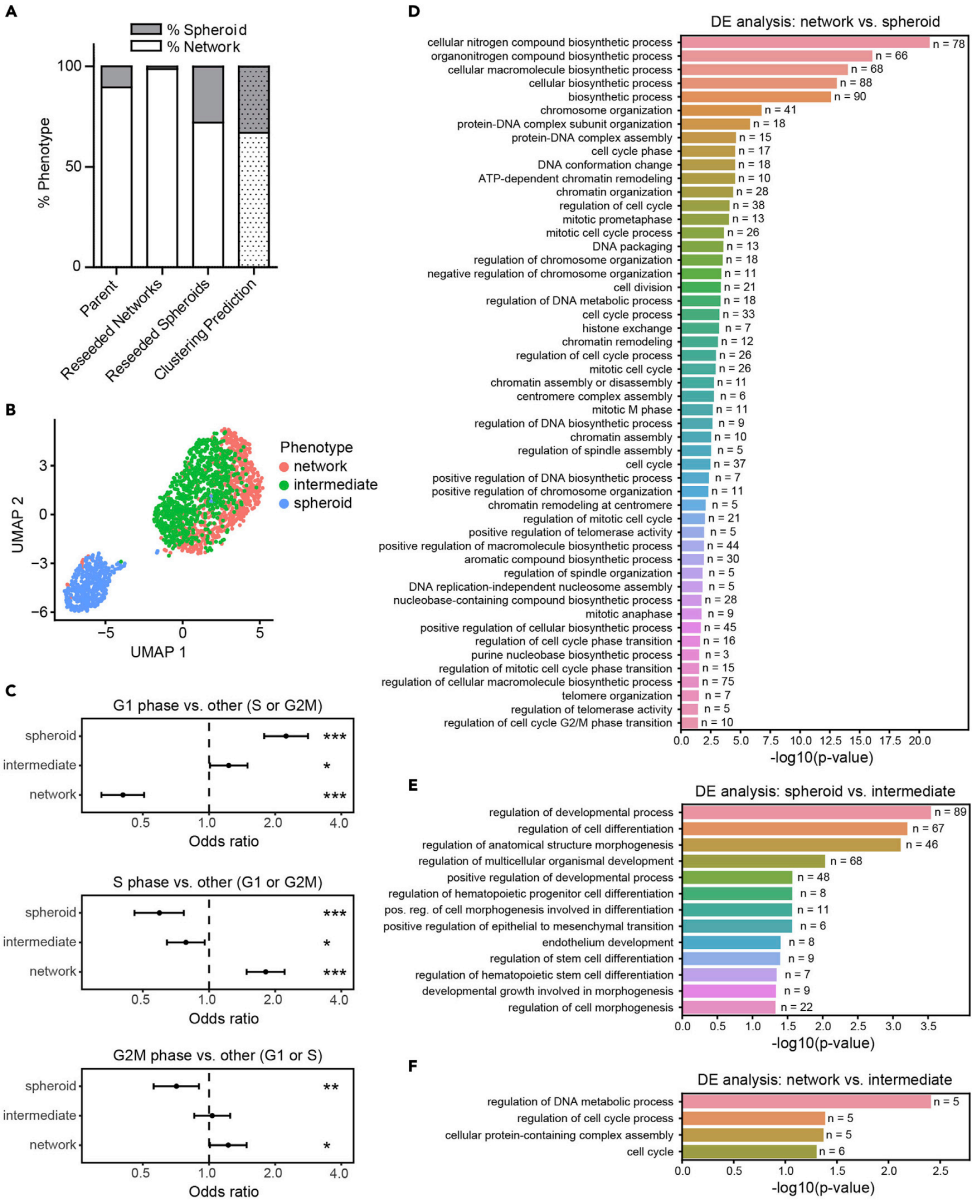
Comparative analysis of phenotypically supervised cell groups to unsupervised cell clusters (steps 62–67) (A) Quantification of spheroid vs. network phenotypes that arise after reseeding from sorted populations. (B) UMAP plot colored by phenotypically supervised cell groups. (C) Cell cycle phase distribution in phenotypically supervised cell groups. Odds ratios (OR) and 95% confidence intervals of G1, S, and G2M cell cycle phases to be prevalent in phenotypically supervised cell groups (spheroid, intermediate or network) using Fisher’s exact test are shown (∗p<0.05, ∗∗p<0.01, ∗∗∗p<0.0001). OR>1 indicates an enrichment of the specified cell cycle phase in the mentioned phenotype, while OR<1 indicates a depletion. (D–F) Barplots showing GO biological processes enriched in genes that are differentially expressed (D) in network cells of cluster 0 compared to spheroid cells; or (E) between spheroid vs. intermediate cells; or (F) in network cells of cluster 0 compared to intermediate cells. Only processes that are associated with differentiation and proliferation are shown. P-values are corrected for multiple testing using the Benjamini & Hochberg method. N represents the number of differentially expressed genes that map to each GO term.

For this dataset, the split of cells labeled as spheroid between unsupervised clusters (Table 4) suggested two states within the spheroid population. In our previous study, we evaluated plasticity of spheroid and network states by sorting and reseeding (Chen et al., 2020). Approximately 75% of reseeded spheroid cells transitioned into network cells, compared to the clustering based prediction of 67%, whereas only about 1% of reseeded network cells formed spheroids after 1 week of culture (Figure 5A). This is consistent with ∼⅔ of spheroid cells clustering with network cells in the single cell data and supports that these may occupy an intermediate state capable of transitioning to the network phenotype. At this point in the analysis, the user must define the number of cell states implicated by combining unsupervised clustering with phenotypic labels. Evidence from scRNAseq and reseeding experiments suggested 3 states: network, spheroid and intermediate (Figure 5B). This was further supported by reanalysis of cell cycle enrichment across the 3 newly defined clusters, which showed that the intermediate spheroid cells had proliferative characteristics that were in between the purely network and spheroid clusters (Figure 5C).

Once a set of cell states is defined, the bioinformatic pipeline implicates biological/functional differences between states using pairwise differential expression analysis between groups. Differentially expressed (DE) genes are identified using DESeq2 (Love et al., 2014) which can be called directly from within Seurat (Stuart et al., 2019), and are annotated with log fold-change, p-value and adjusted p-value (Tables S1, S2, and S3). We identified 650, 410 and 11 DE genes comparing network (in cluster 0) vs. spheroid cells, spheroid vs. intermediate cells, and network (in cluster 0) vs. intermediate cells, respectively. DE genes are further analyzed for gene set enrichment using over-representation analysis (e.g., by Fisher’s Exact Test) using various tools. The pipeline provides instructions for obtaining enriched gene sets using the PANTHER webtool (Mi et al., 2019). Gene set statistics (p-values, gene set sizes and overlapping gene counts) were exported from PANTHER and imported into Python to investigate processes associated with differentiation and proliferation in different cell states (Figures 5D–5F). Network cells were enriched for gene sets associated with proliferation relative to spheroid cells (Figure 5D, Table S4). Although phenotypically similar (i.e., visually spheroid), intermediate cells were distinguished from spheroids by genes involved in differentiation processes such as positive regulation of cell morphogenesis involved in differentiation and positive regulation of epithelial to mesenchymal transition (Figure 5E, Table S5). In contrast, intermediate cells were distinguished from network cells by processes relating to cell cycle (Figure 5F, Table S6). This supports that intermediate cells may be poised to transition out of their spheroid state and raises the possibility that certain genes relating to cell cycle processes may trigger the intermediate phenotype to switch into the more proliferative network phenotype. Table 5 describes the cell differentiation and cell cycle associated genes that distinguish the intermediate cell state from network and spheroid states and could serve as experimental targets for studies related to this hypothesis.

**Table 5:** Selective marker genes distinguishing the intermediate group

Spheroid cells		Intermediate cells		Network cells
DE genes between Spheroid vs. Intermediate			DE genes between Intermediate vs. Network	
annotated for cell differentiation regulation			enriched for cell cycle regulation	
CCL2	MYADM	AC020916	DSTN	CLTC
CDC42EP3	MYO10	CFL1	RPL22	EXOC5
CTNNB1	NRP1	DNM2		GSTP1
CUX1	PDZD8	PSMB2		HIST1H1E
DAB2	PSMA4	PSMD2		HNRNPU
DHX36	PSMD6	RHOC		HSP90AA1
FMNL1	PSME2	S100A10		LBR
FN1	RDX	SDCBP		NPM1
KMT2A	SOX9			PRKDC
LRP10	TGFBR2			
MAP1B	WWTR1			
MAP3K13	YAP1			
MDK	ZMPSTE24			
METRN				

Genes are listed under the cell group in which they are upregulated.

**Figure 6:**
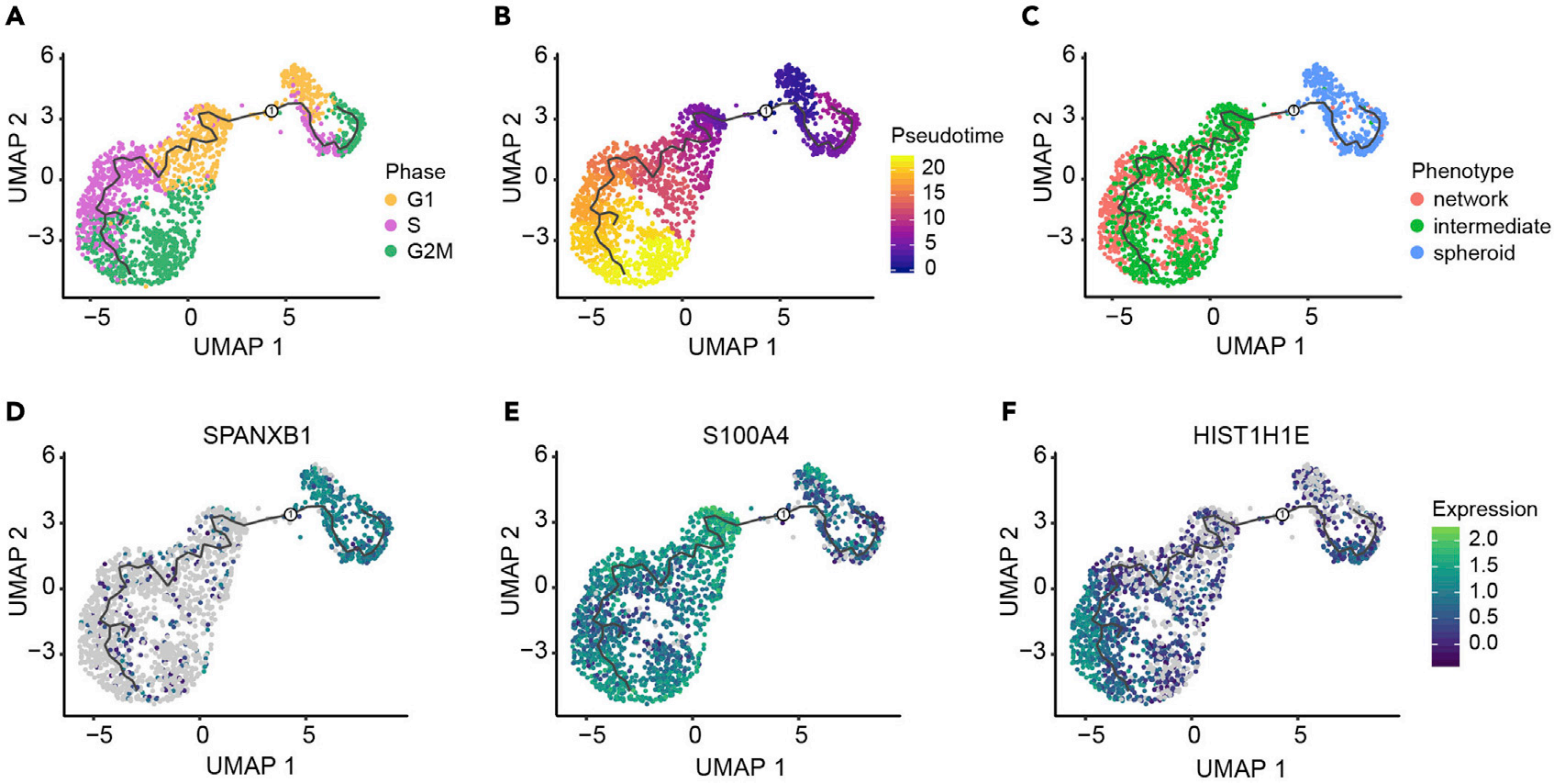
Pseudotime trajectory analysis (steps 68–71) Black lines on the UMAP plots represent the trajectory graph. Root of the pseudotime trajectory is marked as a circle labeled 1. (A–C) UMAP plots with pseudotime trajectories of cells colored by (A) cell cycle phase, (B) pseudotime, and (C) phenotypically supervised cell groups. (D–F) UMAP plots with pseudotime trajectories of cells and the expression of genes (D) SPANXB1, (E) S100A4, and (F) HIST1H1E. Expression is log10 based.

The pipeline provides two additional single cell analyses to aid interpretation of cell states obtained from integrating unsupervised clustering with phenotypic labels. First, pseudotime analysis is used to assess the progressive relationship between cell states. In order to track changes of the cells over time, pseudotime progresses along the trajectory of gene expression changes present in the underlying data starting from a root representing the beginning of a biological process such as differentiation or cell cycle (Cao et al., 2019). The root must be manually selected by the user. As we previously discovered cell cycle association between our phenotypes which could be relevant to cellular transitions (Figures 4E and 5C), we allowed cell cycle effects to be present in the data for pseudotime analysis and selected the midpoint of cells in the G1 cell cycle phase as the root (Figure 6A) to generate pseudotime trajectories (Figure 6B). In this case, the learned trajectories connect spheroid cells to network cells through the intermediate cells (Figure 6C) following cell cycle progression (Figure 6A). DE genes can be visualized along the pseudotime trajectory to obtain higher resolution information about the timing of their expression during transitions (Cao et al., 2019). As a demonstration, we plot DE genes associated with each state: SPANXB1 which is more expressed in spheroid cells (Figure 6D), S100A4 which is more expressed in intermediate cells (Figure 6E), and HIST1H1E which is more expressed in network cells (Figure 6F), specifically compared to the intermediate cells (Table 5).

**Figure 7:**
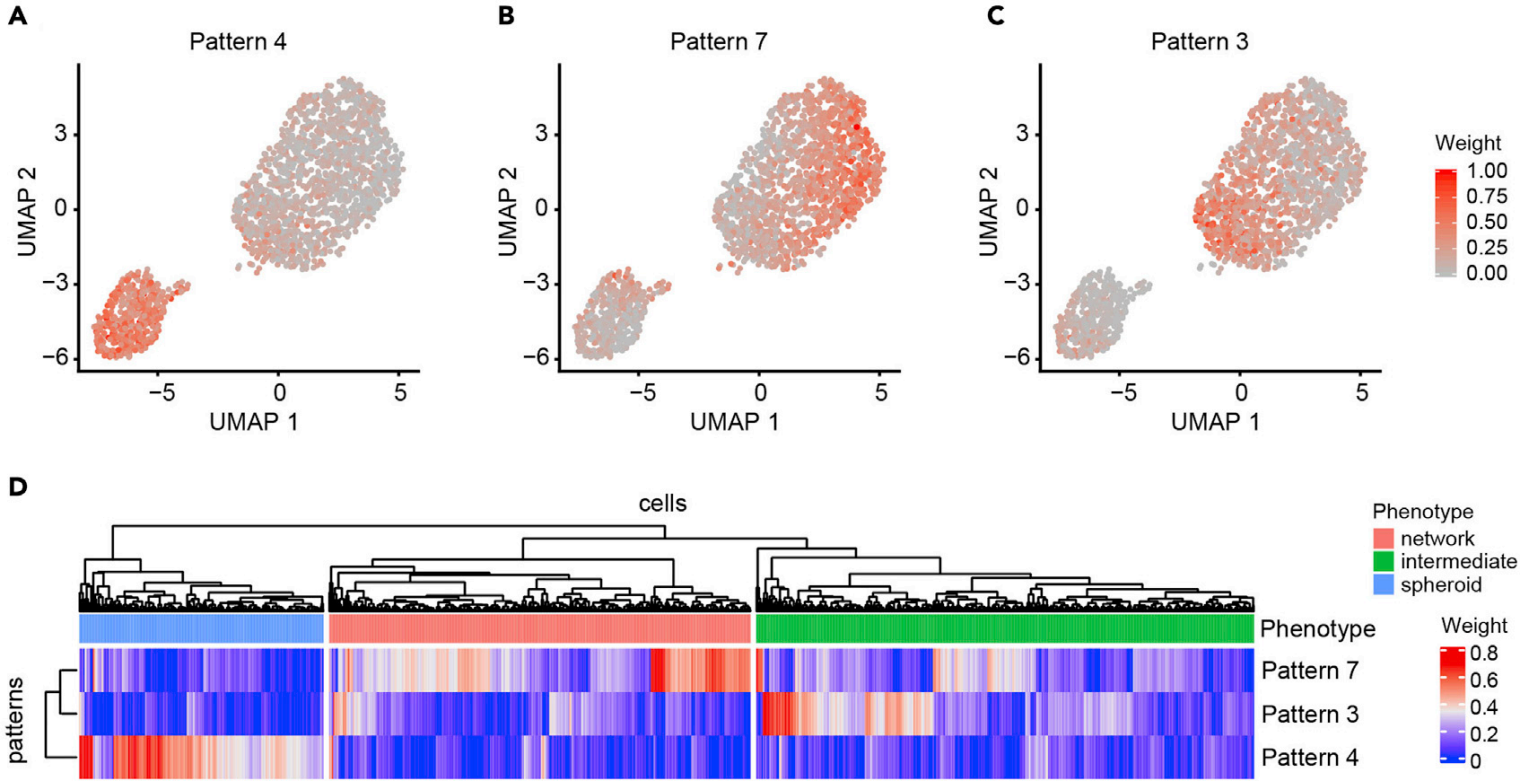
Unsupervised detection of gene expression patterns (steps 72–77) (A–C) UMAP plots overlaid with pattern weights for each cell for the top 3 significant patterns among 7 that best explains the 3 underlying phenotypes (spheroid, intermediate, and network): (A) pattern 4, (B) pattern 7, and (C) pattern 3. Cell cycle phase effects are regressed out. (D) Heatmap of pattern weights by cells for the top 3 significant patterns. Hierarchical clustering is performed on both rows and columns. Clustering on columns is done first between cells within each phenotype, and then between phenotypes. Phenotype annotation is displayed as a bar on top of the heatmap.

**Figure 8:**
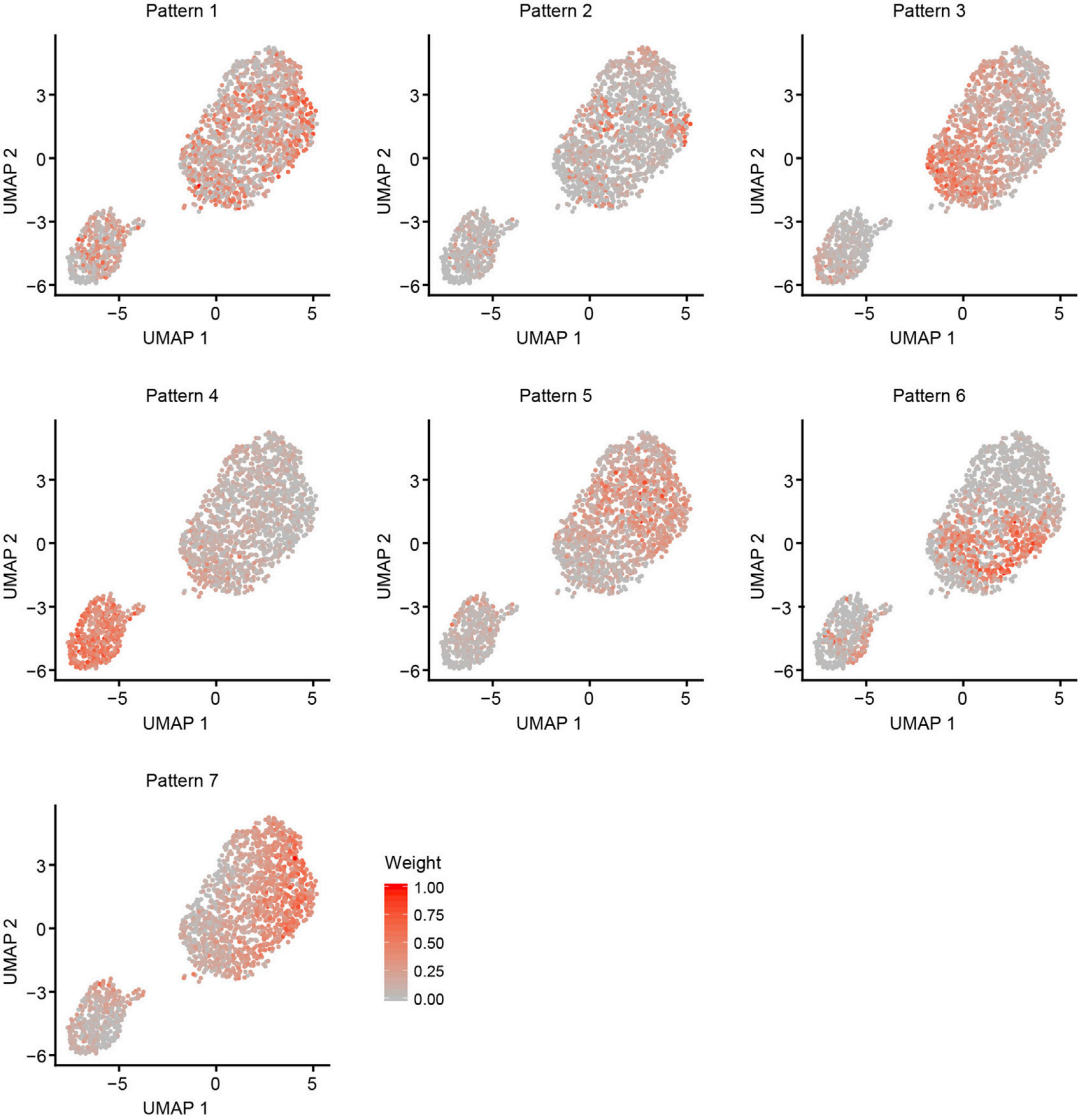
UMAP plots overlaid with pattern weights for each cell for each gene expression pattern (steps 72–75)

**Figure 9:**
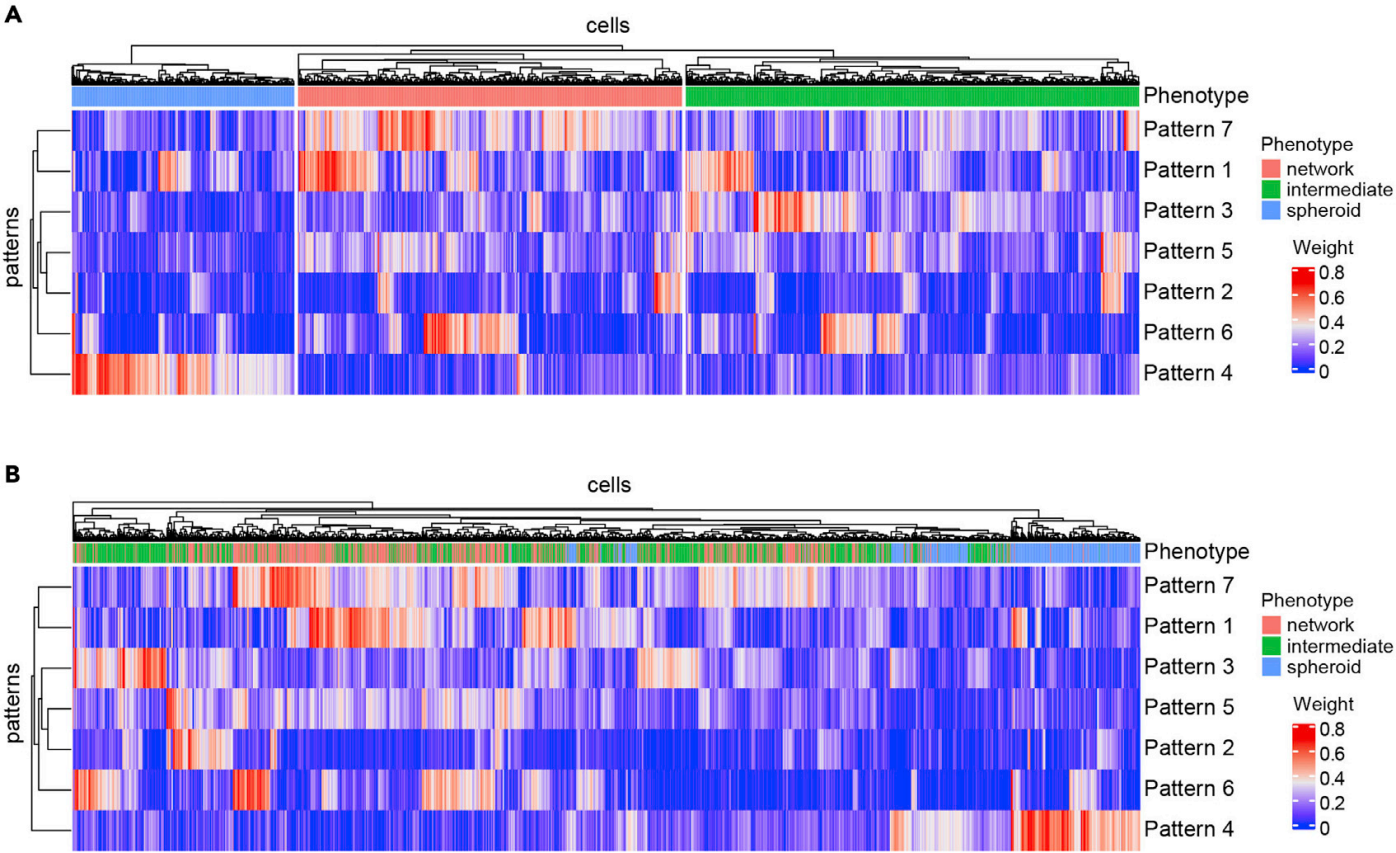
Heatmap of pattern weights by cells for all 7 gene expression patterns (step 77) Hierarchical clustering is performed on both rows and columns. Clustering on columns is done (A) first between cells within each phenotype, and then between phenotypes; or (B) between all cells. Phenotype annotation is displayed as a bar on top of the heatmap.

**Figure 10:**
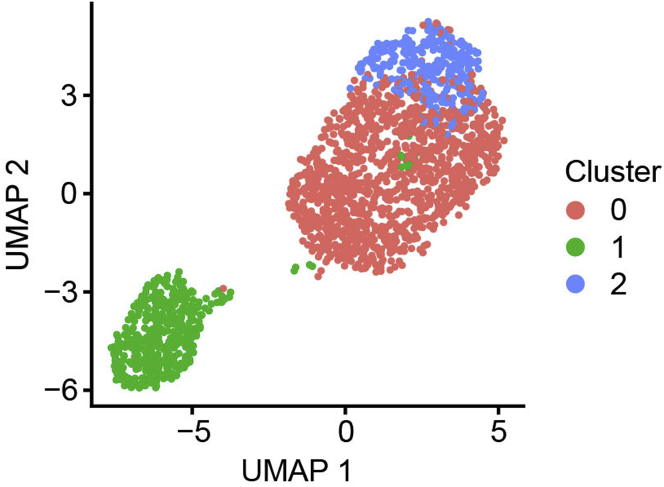
UMAP plot colored by unsupervised clustering into 3 groups

As a final step, the pipeline annotates gene expression programs active in single cells using non-negative matrix factorization (NMF) as implemented by CoGAPS (Fertig et al., 2010). In contrast to performing overrepresentation analysis of DE genes with pre-defined genesets, this approach infers active processes based on latent substructure in the data. The resulting weighted lists of genes associated with each program can be used to gain insight into functional differences across groups of cells. We applied CoGAPS to infer 7 active gene expression patterns in our dataset. Among these, three patterns best explained the expression profiles of spheroid vs. intermediate vs. network phenotypes (Table S7). Pattern 4 detected spheroid cells (Kruskal-Wallis rank-sum test p-value=9.35e-203) (Figure 7A), pattern 7 detected network cells (Kruskal-Wallis rank-sum test p-value=1.96e-141) (Figure 7B), and pattern 3 detected intermediate cells (Kruskal-Wallis rank-sum test p-value=2.43e-115) (Figure 7C), among all 7 patterns (Figure 8). A heatmap of pattern weights further demonstrated the enrichment of pattern 4 for spheroid cells, pattern 7 for network cells and pattern 3 for intermediate cells (Figures 7D and 9). Notably, increasing the number of clusters inferred by Seurat does not detect these patterns (Figure 10), supporting application of both approaches to gain biological insight.

### Applications of the method

Here and in our prior study (Chen et al., 2020), we applied this technique to probe heterogeneity in the collective migration behaviors of MDA-MB-231 breast epithelial cells. Nonetheless, any other aspect of biological heterogeneity can also be investigated using the same approach, provided the heterogeneity manifests in a visual manner. For instance, this system can be used to tackle spatial heterogeneity through the isolation of cells in distinct locations. Time dynamics can also be incorporated by tracking cells and isolating those moving faster from those moving slower. Other extracellular matrices may also be used as part of the culture platform as long as cell recovery strategies are adapted appropriately. Because our fluorescent tagging method is based on transduction of Dendra2, any cell amenable to viral transduction can be used with our method. In addition, the development of Dendra2 mice and intravital microscopy make it potentially possible for our approach to be extended to *in vivo* biology as well.

Combined with downstream FACS and transcriptome sequencing, our photolabeling approach generates insight into the mechanisms of the heterogeneous property in question. This can then inform functional perturbations at the protein level to confirm the inferences made from sequencing outputs. Importantly, in our previous study, gene sets derived using phenotypic metadata differed significantly from those derived from unsupervised analyses alone and enabled the design of more relevant validation experiments. Such phenotype-supervised gene sets can also reduce the number of perturbations required for a full screen of hits compared to unsupervised analysis (near 3-fold reduction from 528 to 178) and uncover unique biomarkers of the phenotypes being studied (Chen et al., 2020).

## Limitations

To increase throughput, our platform uses a wide-field fluorescent microscope outfitted with a laser and galvanometer scanner to photoconvert cells. While the use of a laser maintains x-y spatial resolution, wide-field fluorescence results in a loss in z-spatial resolution compared to confocal scanning and could potentially lead to unintended photoconversion of nearby cells above and below the plane where the targeted cells lie. However, this limitation is overcome by avoiding photoconversion of cells that contain nearby neighbors, and by more sparsely seeding cells in 3D culture to minimize overlap of cells in the vertical axis. With our system, we calculated that objects farther than 10 μm in x-y and farther than 200 μm in z do not receive sufficient exposure to 405 nm light to be photoconverted. We also validated this experimentally.

In our experience, the photoconverted Dendra2 signal is only retained for a maximum of 8 h. For optimal cell recovery, photoconversion and cell isolation should be completed within that time frame. If more time is needed to harvest enough cells, we suggest pooling multiple samples instead of holding a single, long photoconversion session.

## Troubleshooting

### Problem 1

Low transduction efficiency (step 4), possibly caused by low viral titer.

### Potential solution

Increase viral load, or sort cells for expression by flow cytometry and perform growth-based amplification.

### Problem 2

Low fluorescence of photoconverted cells (step 24), possibly caused by low expression of Dendra2 or low photoconversion efficiency.

### Potential solution

Flow sort for the highest Dendra2 expressing cells prior to photoconversion or increase the dwell time/power of the laser.

### Problem 3

Low cell viability post sorting (step 43), possibly caused by high shear stress during cell sorting.

### Potential solution

Reduce the sorting rate on the flow sorter during cell sorting.

### Problem 4

Computational steps cannot be implemented, fail to run, or are time-consuming (steps 49–77), possibly caused by limited computational resources, missing dependencies, or failed software installation.

### Potential solution

Use the provided notebook that runs remotely on the GenePattern webserver.

### Problem 5

Gene expression pattern analysis takes a long time to complete (step 73), possibly caused by the dataset being too large.

### Potential solution

Run the analysis only on a subset of the count matrix using top variable genes, and/or decrease the number of iterations.

## Resource availability

### Lead contact

Further information and requests for resources and reagents should be directed to and will be fulfilled by the lead contact, Stephanie Fraley (sifraley@ucsd.edu).

### Materials availability

This study did not generate new unique reagents.

### Data and code availability

**Figure 11:**
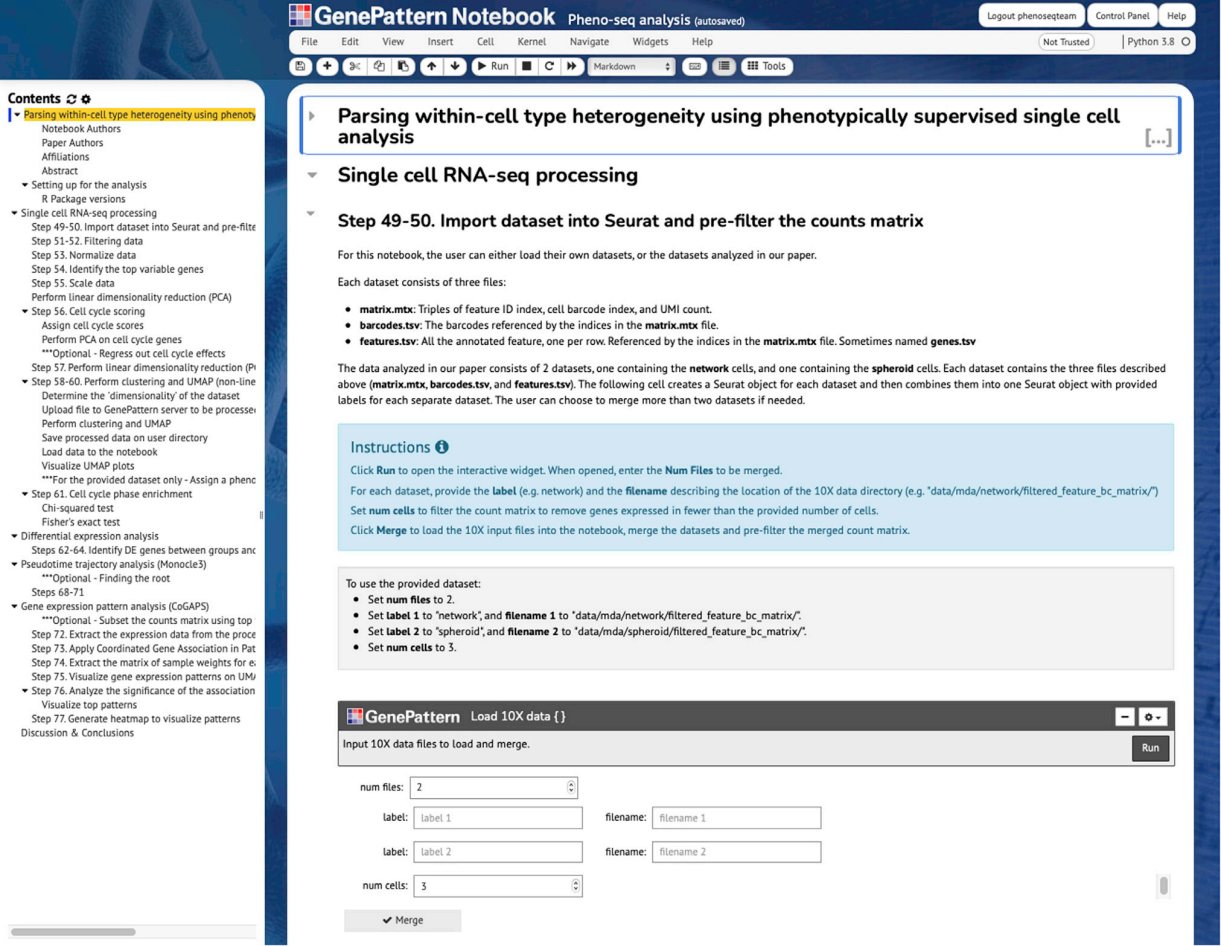
Preview of the GenePattern notebook implementation (steps 49–77) Computational analyses described in the Expected Outcomes section are implemented in a GenePattern notebook (http://notebook.genepattern.org) which can be run on any scRNAseq data by the user or on the dataset analyzed in this paper provided at GEO Series accession number (GSE158844).

Computational analyses described between steps 49–64 and 68–77 in the step-by-step method details section are implemented in a GenePattern notebook (Reich et al., 2017). This notebook can be run on any scRNAseq data by the user or on the dataset analyzed in this paper. The analyzed dataset is provided at the National Center for Biotechnology Information Gene Expression Omnibus (GEO) as a supplementary file named “GSE158844_MDA_10X_output.tar.gz,” and the accession number for this dataset is GEO: GSE158844 (https://www.ncbi.nlm.nih.gov/geo/query/acc.cgi?acc=GSE158844). In order to use the notebook, log in to the GenePattern notebook server, http://notebook.genepattern.org. In the Notebook Library, select the “Pheno-seq analysis” notebook and choose Run. In the notebook, procedure steps are grouped according to task and can be run sequentially as is, or parameters and code can be modified to accommodate variants of the workflow (Figure 11).

## Supplemental information

Table S1. Differential expression analysis between network (in cluster 0) vs. spheroid cells, related to steps 62–64 Among 650 DE genes, 268 are upregulated in network cells, while 382 are upregulated in spheroid cells. For each gene, log fold-change (avg_logFC), fraction of network (pct.1) or spheroid (pct.2) cells where gene is detected, raw p-value, and adjusted p-value using Benjamini & Hochberg method (p_value_adj_BH) are provided. A negative log fold-change indicates higher expression in spheroid cells, while a positive log fold-change indicates higher expression in network cells.

Table S2. Differential expression analysis between spheroid vs. intermediate cells, related to steps 62–64 Among 410 DE genes, 307 are upregulated in spheroid cells, while 103 are upregulated in intermediate cells. For each gene, log fold-change (avg_logFC), fraction of intermediate (pct.1) or spheroid (pct.2) cells where gene is detected, raw p-value, and adjusted p-value using Benjamini & Hochberg method (p_value_adj_BH) are provided. A negative log fold-change indicates higher expression in spheroid cells, while a positive log fold-change indicates higher expression in intermediate cells.

Table S3. Differential expression analysis between network (in cluster 0) vs. intermediate cells, related to steps 62–64 Among 11 DE genes, 9 are upregulated in network cells, while 2 are upregulated in intermediate cells. For each gene, log fold-change (avg_logFC), fraction of network (pct.1) or intermediate (pct.2) cells where gene is detected, raw p-value, and adjusted p-value using Benjamini & Hochberg method (p_value_adj_BH) are provided. A negative log fold-change indicates higher expression in intermediate cells, while a positive log fold-change indicates higher expression in network cells.

Table S4. GO term enrichment analysis on DE genes between network (in cluster 0) vs. spheroid cells, related to steps 65–67 GO term enrichment analysis of the 268 genes that are upregulated in network cells compared to spheroid cells. For each term, total gene set size, the number of DE genes that map to the set (overlap), fold enrichment, raw p-value, and false discovery rate (fdr) as calculated by the Benjamini & Hochberg method are provided.

Table S5. GO term enrichment analysis on DE genes between spheroid vs. intermediate cells, related to steps 65–67 GO term enrichment analysis of the 410 genes that are differentially expressed between spheroid and intermediate cells. For each term, total gene set size, the number of DE genes that map to the set (overlap), fold enrichment, raw p-value, and false discovery rate (fdr) as calculated by the Benjamini & Hochberg method are provided.

Table S6. GO term enrichment analysis on DE genes between network (in cluster 0) vs. intermediate cells, related to steps 65–67 GO term enrichment analysis of the 9 genes that are upregulated in network cells compared to intermediate cells. For each term, total gene set size, the number of DE genes that map to the set (overlap), fold enrichment, raw p-value, and false discovery rate (fdr) as calculated by the Benjamini & Hochberg method are provided.

Table S7. CoGAPS patterns ranked by p-value using Kruskal-Wallis rank-sum test, related to steps 72–77 The test evaluates the distribution of pattern weights across 3 phenotypes: spheroid vs. intermediate vs. network cells. Test statistic, p-value, and adjusted p-value using Benjamini & Hochberg method (p_value_BH) for each pattern is provided.
